# A wearable nanoscale heart sound sensor based on P(VDF-TrFE)/ZnO/GR and its application in cardiac disease detection

**DOI:** 10.3762/bjnano.14.67

**Published:** 2023-07-31

**Authors:** Yi Luo, Jian Liu, Jiachang Zhang, Yu Xiao, Ying Wu, Zhidong Zhao

**Affiliations:** 1 School of Electronics and Information Engineering, Hangzhou DIANZI University, Hangzhou 310018, China; 2 School of Communication Engineering, Hangzhou DIANZI University, Hangzhou 310018, China; 3 Academic Affairs Office, Hangzhou DIANZI University, Hangzhou 310018, China; 4 School of Cyberspace Security, Hangzhou DIANZI University, Hangzhou 310018, China

**Keywords:** composite piezoelectric nanofilm, electrospinning, heart sound classification algorithm, heart sound sensor, heart sound stethoscope

## Abstract

This paper describes a method for preparing flexible composite piezoelectric nanofilms of P(VDF-TrFE)/ZnO/graphene using a high-voltage electrospinning method. Composition and β-phase content of the piezoelectric composite films were analyzed using X-ray diffraction. The morphology of the composite film fibers was observed through scanning electron microscopy. Finally, the P(VDF-TrFE)/ZnO/graphene composite film was encapsulated in a sandwich-structure heart sound sensor, and a visual heart sound acquisition and classification system was designed using LabVIEW. A heart sound classification model was trained based on a fine *K*-nearest neighbor classification algorithm to predict whether the collected heart sounds are normal or abnormal. The heart sound detection system designed in this paper can collect heart sound signals in real time and predict whether the heart sounds are normal or abnormal, providing a new solution for the diagnosis of heart diseases.

## Introduction

According to data released by the World Health Organization (WHO), approximately 17.9 million people die each year from cardiovascular diseases (CVDs) [1]. CVDs have always been one of the primary diseases affecting human health. In recent years, the number of cardiovascular patients has continued to increase. Heart sounds are physiological signals generated by the movement of heart valves, myocardium, blood, and other parts of the heart. They provide a significant amount of information about the heart and blood vessels [2]. Therefore, cardiac auscultation is considered one of the most effective methods for diagnosing CVDs.

Cardiac auscultation usually requires the aid of a stethoscope. In 1816, the French physician Laennec invented the stethoscope, which was gradually improved to form the current acoustic stethoscope [3]. A traditional acoustic stethoscope is composed of three parts, namely auscultation head, sound guide hose, and ear hook. It uses the principle of physical sound transmission to collect and transmit the heart sound. In order to overcome the shortcomings of the acoustic stethoscope, which lacks amplification during auscultation, is susceptible to noise, and relies heavily on the practitioner’s experience, new stethoscope technologies have emerged as contemporary research fields, including electronic stethoscopes [4–5], Doppler stethoscopes [6–7], and Bluetooth heart sound stethoscopes [8]. Piezoelectric materials, which can convert mechanical vibration signals into voltage signals, have become one of the primary materials for creating heart sound sensors [9].

Piezoelectric materials are essential components in heart sound auscultation equipment. When pressure is applied to piezoelectric materials, they generate a voltage, a phenomenon known as the positive piezoelectric effect. Most current electronic stethoscopes utilize the positive piezoelectric properties of rigid piezoelectric materials such as lead zirconate titanate (Pb(Zr_1−_*_x_*Ti*_x_*)O_3_, PZT). These materials convert sound wave vibrations into proportional electrical signals. After a series of processing steps, the heart sound signal is obtained. However, PZT has a brittle texture, does not fit the skin well, and lacks comfort when worn, making it unsuitable for wearable sensors [10]. Moreover, the lead in PZT is harmful to humans. In recent years, there has been a significant advancement in wearable electronic devices within the healthcare field, leading to several noteworthy breakthroughs. For instance, D. Wan et al. [11] presented a groundbreaking development in the form of a flexible wearable friction patch. This innovative patch consists of a dual-layer PDMS membrane infused with hydrogel. It harnesses the energy generated from bodily movements and utilizes it to create an electric field between the friction patch and the surrounding body tissues, thereby promoting the expedited healing of wounds. Poly(vinylidene fluoride–trifluoroethylene) (P(VDF-TrFE)) is a piezoelectric polymer material with a wide frequency bandwidth, good biocompatibility, and softness. It is one of the preferred materials for flexible piezoelectric sensors [12]. However, compared to rigid piezoelectric materials such as PZT, pure P(VDF-TrFE) has inferior piezoelectric properties [13]. Researchers have improved the film-making process by adding fillers to P(VDF-TrFE), using secondary polarization, and applying other methods to enhance its piezoelectric performance. Kumar et al. prepared P(VDF-TrFE)/ZnO matrix composite nanogenerators using electrospinning. Voltage and current of these nanogenerators were, respectively, 2.4 times and 1.6 times greater than those of pure P(VDF-TrFE) nanogenerators [14]. Subash et al. added ZnO nanoparticles and exfoliated graphene oxide to P(VDF-TrFE) to prepare a composite nanofilm with excellent touch sensitivity and high output energy. They also used the piezoelectric film for energy harvesting [15].

Applying machine learning classification algorithms in the domain of human physiological signal detection is presently a prominent area of research. A notable study by R. Guo et al. [16] successfully integrated deep learning techniques with frictional hydrogel sensors to achieve comprehensive monitoring of infant movements. Similarly, there is considerable research interest in the classification of heart sound signals and the development of intelligent systems for diagnosing heart diseases. These research directions hold significant importance in contemporary society. The process of analyzing heart sounds mainly involves three parts: signal preprocessing, feature extraction, and classification recognition. The classification methods of heart sound signals can be divided into several types, including BP neural network, support vector machines (SVMs), Gaussian mixture models, wavelet neural network, and hidden Markov model-based and clustering-based methods. For example, Zheng et al. successfully implemented computer-aided diagnosis of chronic heart failure using a least squares SVM [17].

In this paper, to enable real-time monitoring and early detection of cardiovascular diseases, a flexible piezoelectric thin film heart sound sensor was developed, and a heart sound detection and classification system was built based on this sensor. Zinc oxide (ZnO) and graphene (GR) fillers were added to the P(VDF-TrFE) matrix, and P(VDF-TrFE)/ZnO/GR composite piezoelectric nanofilms were prepared using a high-voltage electrospinning process. The resulting piezoelectric nanofilm was encapsulated into a wearable, flexible heart sound sensor for detecting heart sounds. A heart sound detection system was then built using LabVIEW software, which detects heart sounds and extracts features from the preprocessed heart sound signals using MATLAB scripts. Finally, a *K*-nearest neighbor (KNN) heart sound classification model was trained and used to predict whether a heart sound is normal or abnormal, with the prediction result displayed.

## Experimental

### Preparation of films by high-voltage electrospinning

**Figure 1:**
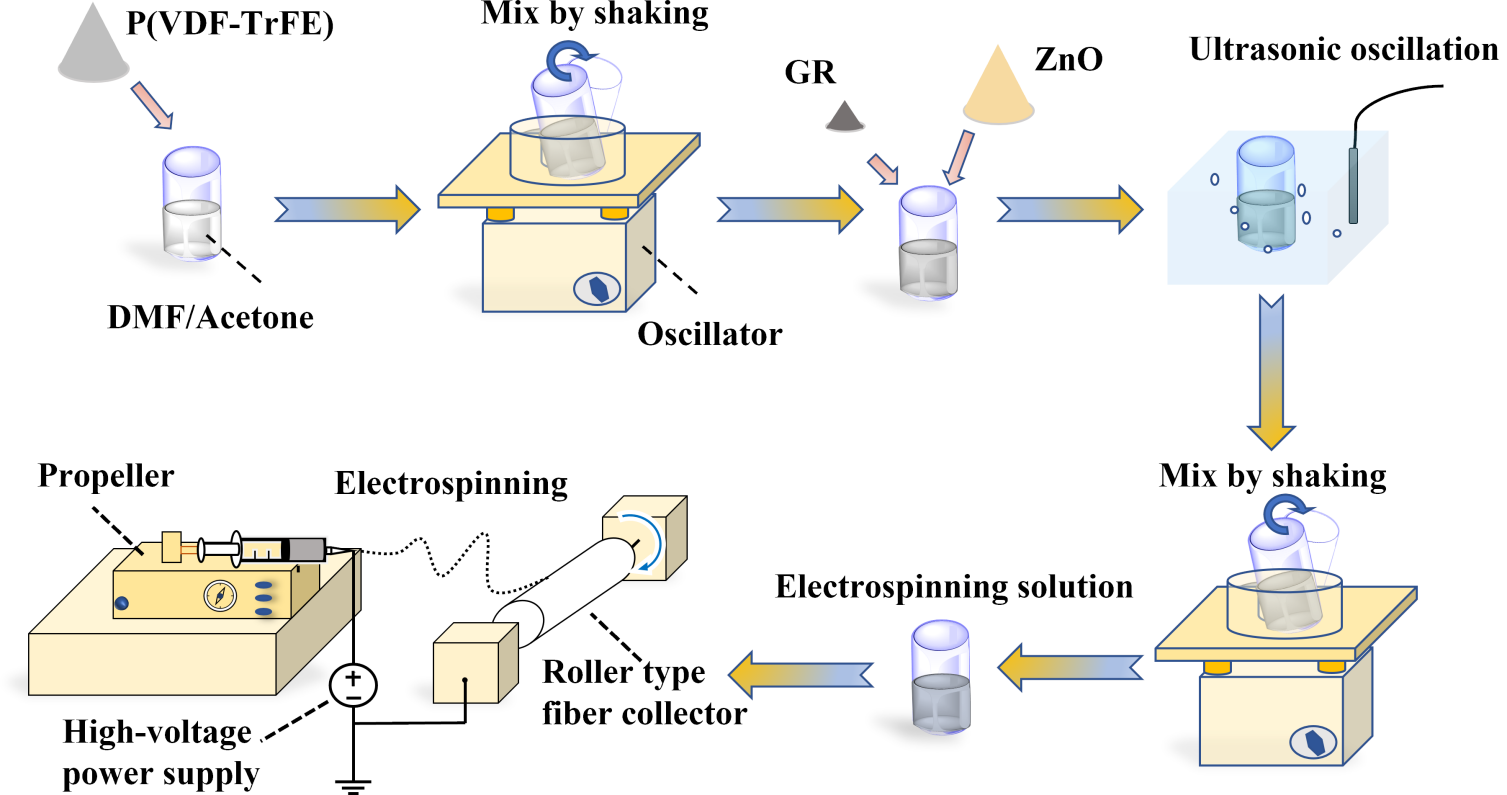
Preparation process of composite piezoelectric nanofilm.

In this paper, composite piezoelectric nanofilms were prepared using a high-voltage electrospinning process. This experimental setup comprises three main components, namely a high-voltage DC power supply, micro-pump spinnerets, and a fiber collector [18]. The piezoelectric nanofilms produced using this process possess flexibility and do not require secondary polarization. Additionally, this technique is cost-effective, convenient, and straightforward, making it an optimal method for preparing piezoelectric nanofilms. The specific methodology used to prepare the piezoelectric nanofilms is illustrated in Figure 1. First, P(VDF-TrFE) powder (Solvay, France, molar ratio 7:3) was mixed with *N*,*N*-dimethylamide (DMF, Acros) and acetone (Sinopharm Chemical Reagent Co., Ltd., evaporation residue <0.001%) in a mixed solution with a 3:2 volume ratio. The mass fraction of P(VDF-TrFE) was 12%. Second, the reagent bottle was sealed and placed in a shaking mixer and shaken for 3 h. Next, ZnO nanoparticles (Shanghai Keyan Industrial Co., Ltd., particle size 3 ± 5 nm, content ≥99.8%) and GR filler (Shenzhen Turing Evolution Technology Co., Ltd., carbon content 98%, average diameter/thickness ratio = 9500) were added to the mixed solution. The mass fraction of ZnO was 10%, and the mass fraction of GR was 0.1%. The particles in the solution were dispersed by ultrasonic treatment for 2 h. Finally, the solution was shaken and mixed for 3 h to obtain the electrospinning solution. The electrospinning solution was aspirated using a 15 mL syringe, placed on a micropump propeller, and propelled at a speed of 1.5 mL/h for high-voltage electrospinning. The voltage was set to 20 kV, and the distance from the needle to the roller collector was set to 13 cm. Ambient temperature and humidity during high-voltage electrospinning were controlled at 25 °C and 40% RH, respectively.

### Fabrication of wearable flexible nanoscale heart sound sensors

**Figure 2:**
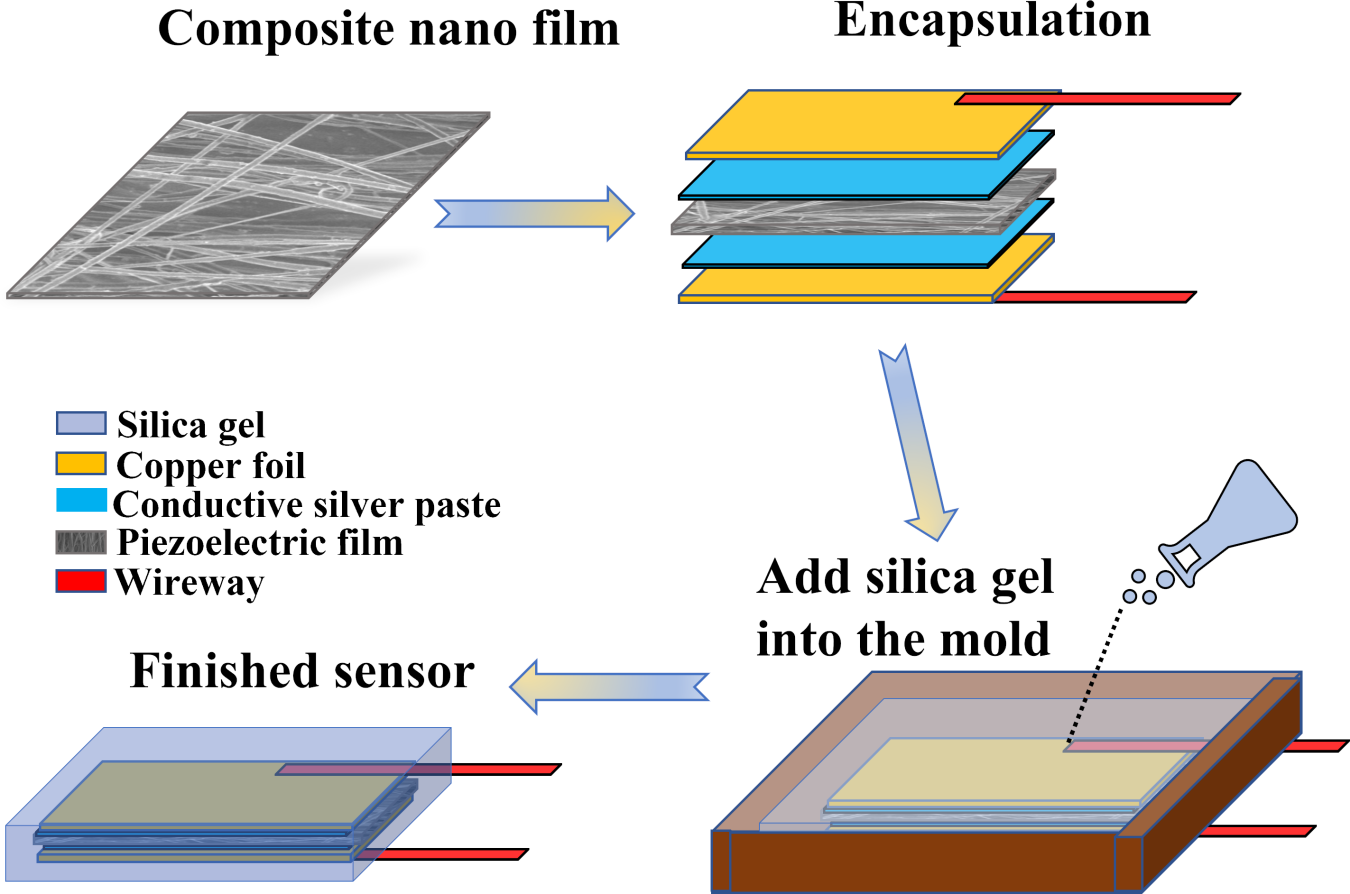
Flexible sensor packaging.

**Figure 3:**
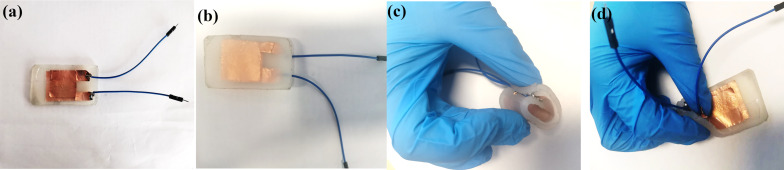
Actual sensor.

Figure 2 illustrates the process of creating a wearable, flexible nanoscale heart sound sensor with a sandwich structure. First, a rectangular composite nanofilm measuring 4.5 cm in length and 3 cm in width is cut, and a layer of conductive silver paste is evenly applied to both sides of the film. The paste is then dried completely by heating on a plate at 55 °C for 2 h. Copper foil tape is used as an electrode to attach to the conductive silver paste layer on both sides, and wires are welded onto the edges of the copper foil. In the mold shown in Figure 2, a thin layer of silica gel is injected and left to stand until it is dry. The nanofilm attached to the electrode is then placed in the mold, and an appropriate amount of silica gel is slowly injected until the film is completely immersed. After letting it stand for two days, the flexible nanoscale heart sound sensor is ready for use. Figure 3 displays the actual sensor produced. Figure 3a and Figure 3b show, respectively, photos of the front and the back side of the sensor, and Figure 3c and Figure 3d show photos of the sensor after bending. It was observed that the sensor encapsulated by silica gel was highly flexible and could fit snugly onto the skin. Additionally, the food-grade silica gel used in the outermost layer is ecologically friendly, harmless to human health, and makes for a great wearable, flexible nanosensor.

### Construction of acoustic-electric conversion test platform

**Figure 4:**
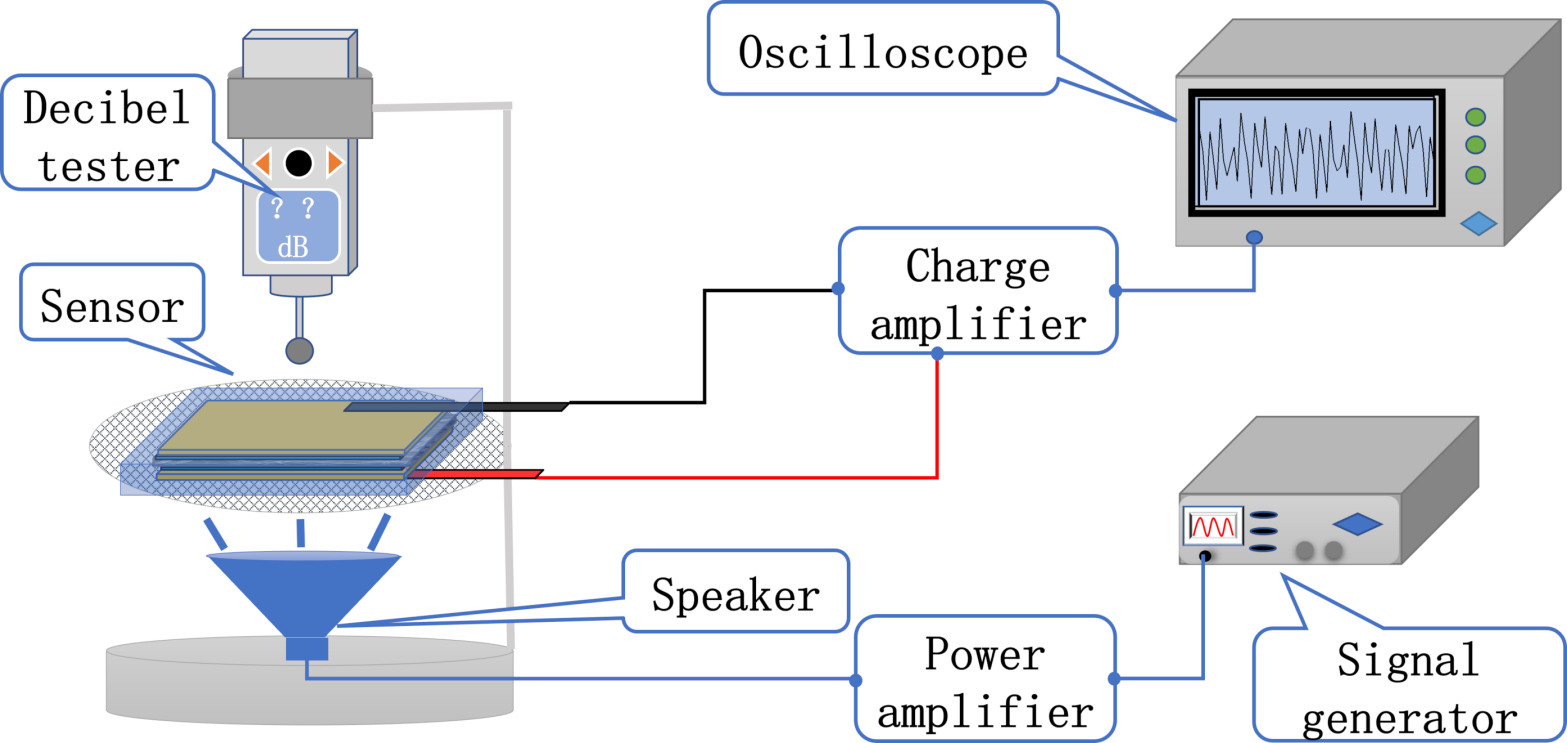
Acoustic-electric conversion performance test platform.

The heart sound sensor needs to convert the signal of the heart sound into an electrical signal, requiring the sensor to meet certain requirements in terms of its acoustic and electrical conversion performance. A custom-built platform (Figure 4) was used to evaluate the acoustic-electric conversion capability of the sensor. The platform consists of an oscilloscope, a charge amplifier, a signal generator, a power amplifier, a speaker, a fixed bracket, and a decibel tester. The signal generator produces a continuous periodic electrical signal, which is then amplified by the power amplifier and delivered to the speaker to convert the electrical signal into an acoustic signal. A flexible nanoscale heart sound sensor is placed 1 cm above the speaker, and its electrodes are connected to the charge amplifier and then to the oscilloscope. The voltage amplitude and frequency displayed on the oscilloscope demonstrate the heart sound sensor's acoustic-electric conversion ability. The loudspeaker's emitted sound frequency is controlled by setting the signal generator to produce a sine wave signal with different frequencies, while the decibel level of the sound emitted is adjusted by regulating the amplitude of the signal generated and the power amplifier's amplification. During the electric-acoustic conversion performance test, a decibel tester is placed 1.5 cm above the heart sound sensor to control the sound pressure.

### Construction of a heart sound acquisition and classification system

**Figure 5:**
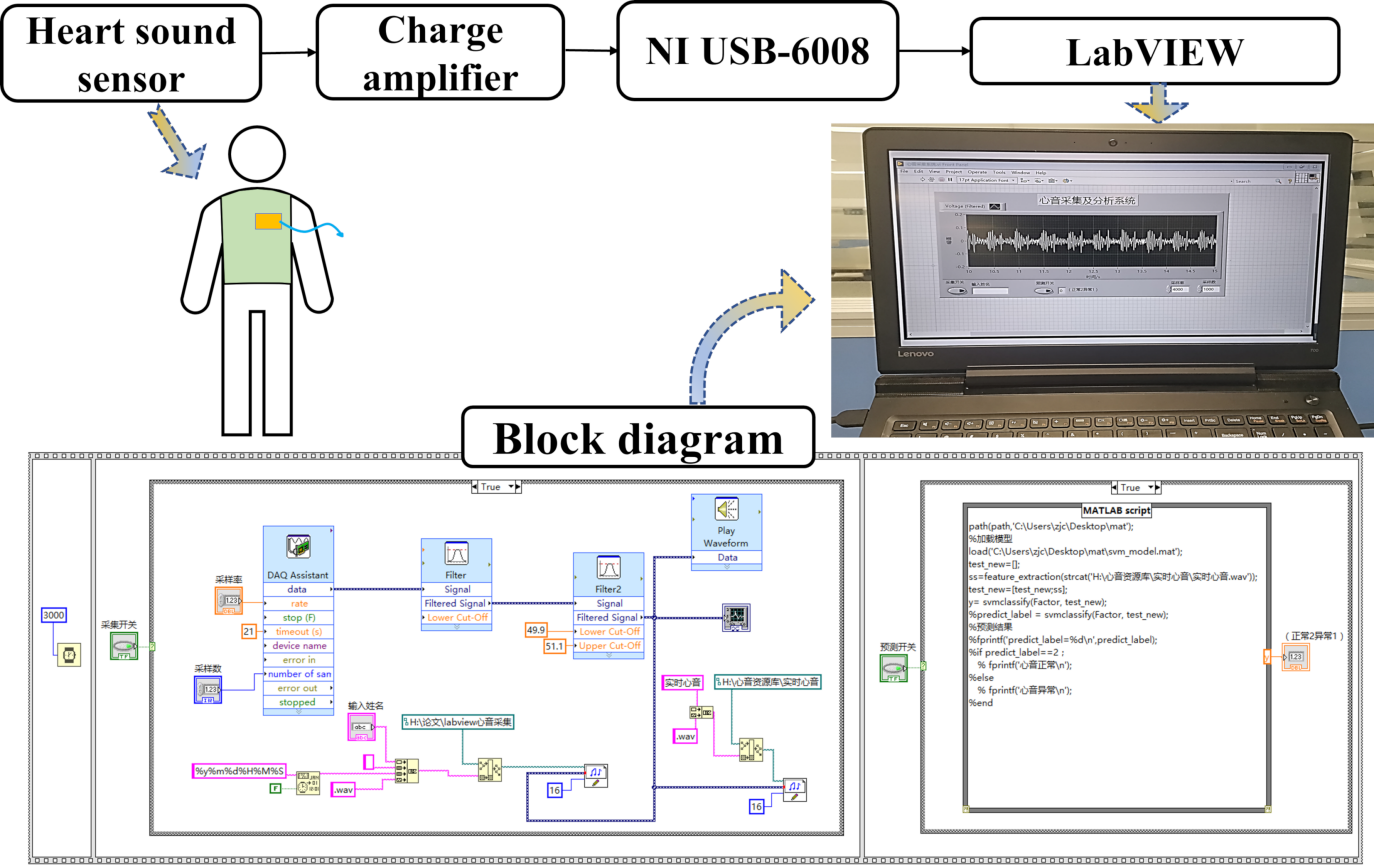
Block diagram of the heart sound acquisition and classification system.

The experiment involved setting up a heart sound acquisition and classification system is illustrated in Figure 5. The experimental flexible nanoscale heart sound sensor was connected to a charge amplifier with a charge amplification set to 100 pC/V. The open-circuit voltage was then measured using the NI USB-6008 data acquisition card and displayed on a computer via LabVIEW software. LabVIEW is a programming environment that utilizes graphical programming language for designing virtual instruments for experiments using the “G” language. The program designed in this article primarily uses the “DAQ Assistant”, which is the driver programming assistant of the NI acquisition card, to receive and process the collected data. During the experiment, the test subjects affixed the heart sound sensor onto the mitral valve auscultation area with medical tape and maintained a sitting posture for approximately 50 s while setting the sampling rate to 2000 Hz. The heart sound acquisition system built in the experiment collected the heart sound signal for 20 s. The acquired heart sound signal was then filtered and denoised by a 20 to 200 Hz band-pass filter and a 50 Hz notch filter, respectively, after which the processed heart sound signal waveform was displayed on the front panel. The band-pass filter of the acquisition system is an eigth-order ellipse filter, while the notch filter is a third-order Butterworth filter. The program also features the function of storing the collected heart sound signal and the function of playing the collected heart sound audio in real time.

LabVIEW enables a combination of graphical programming with the MATLAB language by using a MATLAB script node. In the LabVIEW program developed in this study, real-time heart sound data collected from the acquisition system is fed into a classification model trained in MATLAB. The program then predicts whether the heart sound is normal or abnormal based on the model, and displays the prediction results on the front panel.

By observing the waveform displayed on the front panel, it is evident that the signal filtered by the two filters exhibits distinct characteristics of the first and second heart sounds. While observing the heart sound waveform, the collected heart sound can be heard via connected headphones. The combination of audio and visual cues can more accurately differentiate normal or abnormal heart sound signals. Real-time collected heart sound signals are fed into the *K*-nearest neighbor (KNN) classification model for prediction. After waiting for approximately 5 s, the prediction result is displayed on the front panel as a numeric value: “1” indicates abnormal heart sound, while “2” indicates normal heart sound. In general, this heart sound acquisition system can quickly collect and predict heart sound signals, while also providing audio and visual output. The heart sound acquisition probe is soft, comfortable, and advanced, with the potential to become a new type of heart sound stethoscope.

## Results and Discussion

### Characterization of thin film samples

**Figure 6:**
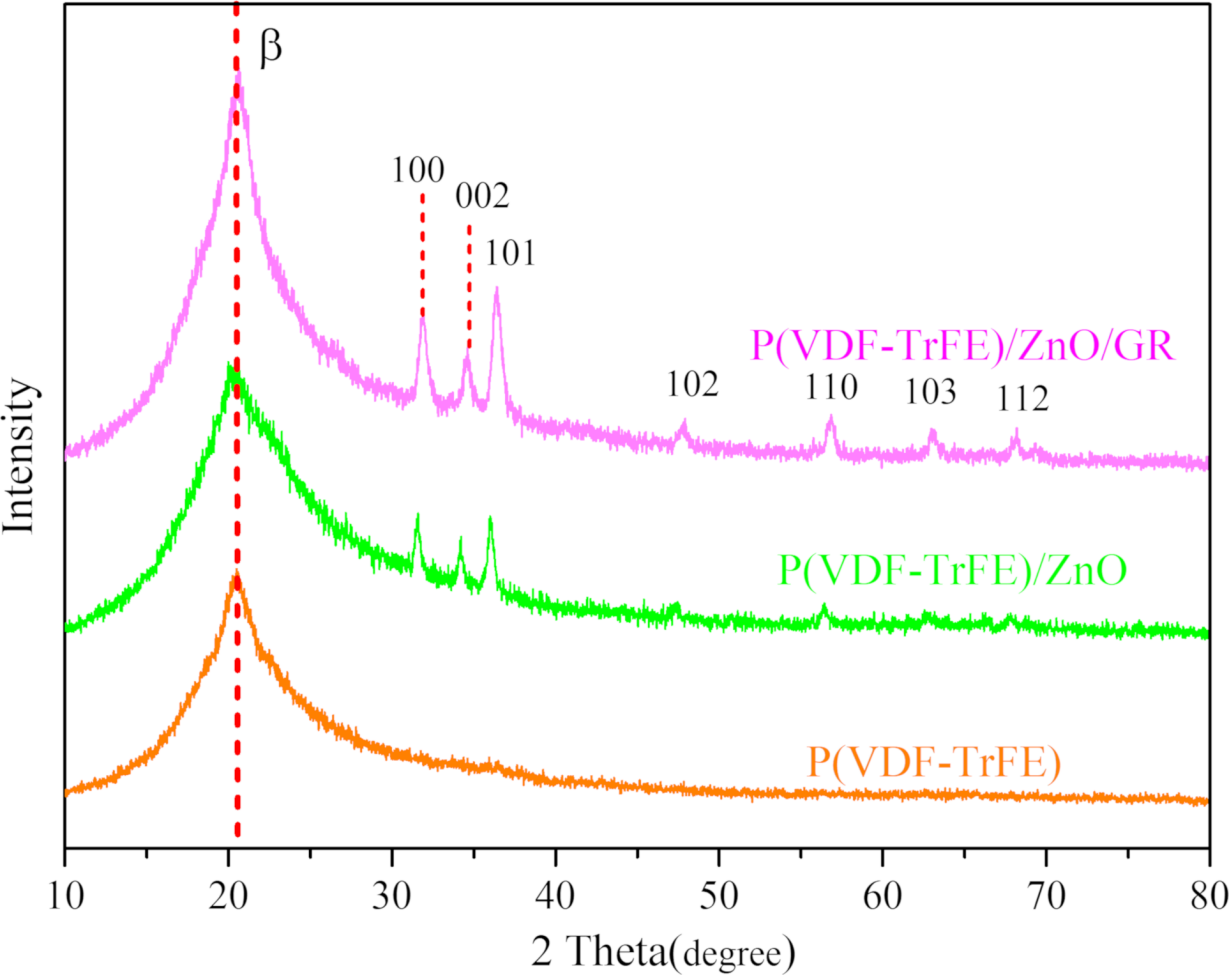
XRD patterns of the three composite piezoelectric nanofilms.

In the study by Luo et al. [19], an investigation was carried out to explore the electrospinning of piezoelectric films using different composition ratios of P(VDF-TrFE), ZnO, and GR. The resulting films underwent characterization through electron microscopy, X-ray diffraction (XRD), and piezoelectric performance testing. The results indicated that the piezoelectric film with a composition ratio of 12% P(VDF-TrFE) + 10% ZnO + 0.1% GR exhibited superior performance regarding various aspects. Consequently, in this present study, we employed the aforementioned composition ratio to fabricate the heart sound sensor. Our paper solely focuses on the performance evaluation of three experimental groups: 12% P(VDF-TrFE), 12% P(VDF-TrFE) + 10% ZnO, and 12% P(VDF-TrFE) + 10% ZnO + 0.1% GR. In this paper, XRD was utilized to analyze the composition and β-phase content of the composite piezoelectric nanofilms, while scanning electron microscopy (SEM) was employed to observe the morphology of the thin film filaments. Figure 6 displays the XRD patterns of the three composite piezoelectric nanofilms. In the P(VDF-TrFE)/ZnO film, the mass fraction of ZnO is 10%, while in the P(VDF-TrFE)/ZnO/GR film, the mass fractions of ZnO and GR are 10% and 0.1%, respectively. The addition of ZnO resulted in the appearance of seven characteristic reflections, namely (100), (002), (101), (102), (110), (103), and (112) of ZnO on the XRD map of the composite nanofilm. This indicates that ZnO exists in the form of nanoparticles in the fiber film after being added to P(VDF-TrFE) [20–21].

P(VDF-TrFE)/ZnO/GR exhibits the highest β-phase content among the three films, with P(VDF-TrFE)/ZnO showing a slightly higher content than P(VDF-TrFE). Graphene (GR) exhibits outstanding electrical conductivity and strong adsorption capabilities. When combined with ZnO, it forms a more homogeneous and compact structure, thereby enhancing the alignment and arrangement of ZnO crystals. Furthermore, the layered structure of GR enables it to enwrap the ZnO particles, leading to stronger reflections of ZnO in the XRD pattern. However, an excessive addition of GR may lead to the formation of larger particles or agglomerates of GR. Consequently, this can compromise the alignment and arrangement of the ZnO crystals, thereby weakening the lattice structure of ZnO and ultimately leading to a reduction in the intensity of the ZnO reflections in the XRD pattern [22]. Experimental results suggest that adding ZnO and trace amounts of GR filler can increase the β-phase content of the composite piezoelectric film [23–25]. A higher β-phase content is generally associated with better piezoelectric performance of the material.

**Figure 7:**
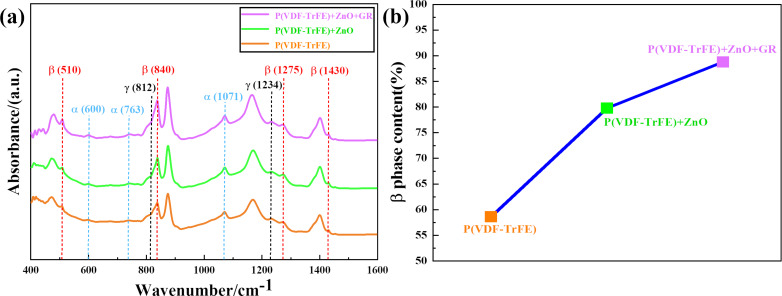
(a) FTIR spectrum and (b) variation curve of β-phase content.

To further validate the enhancement of the β phase, Fourier-transform infrared (FTIR) analysis was conducted (Figure 7a). The FTIR spectrum of the piezoelectric film exhibited distinct absorption bands. Peaks observed at 600, 763, and 1071 cm^−1^ correspond to the α-phase [26], whereas the γ-phase peaks were observed at 812 cm^−1^ [27–28] and 1234 cm^−1^ [29]. The polar β-phase was characterized by the absorption bands at 510, 840, 1275, and 1430 cm^−1^ [30]. From the spectrum, it was evident that the β-phase in the P(VDF-TrFE) + ZnO + GR group exhibited an enhancement compared to the other two groups at 510, 840, 1275, and 1430 cm^−1^, which correlated with the XRD findings. The specific value of the β-phase content (*F*(β)) can be determined using the Lambert–Beer law [31]:


[1] $$F\left(\beta \right)=\frac{A_{\beta }}{A_{\alpha }\cdot \frac{K_{\alpha }}{K_{\beta }}+A_{\beta }},$$

where *A*_α_ and *A*_β_ represent the absorbance intensities at 762 and 840 cm^−1^, respectively, and *K*_α_ and *K*_β_ are the absorption coefficients with values of 6.1 × 10^4^ cm^2^/mol and 7.7 × 10^4^ cm^2^/mol, respectively [31]. The calculation results are depicted in Figure 7b, demonstrating a notable enhancement in the β-phase content upon the incorporation of ZnO and GR, which is in agreement with the XRD findings.

**Figure 8:**
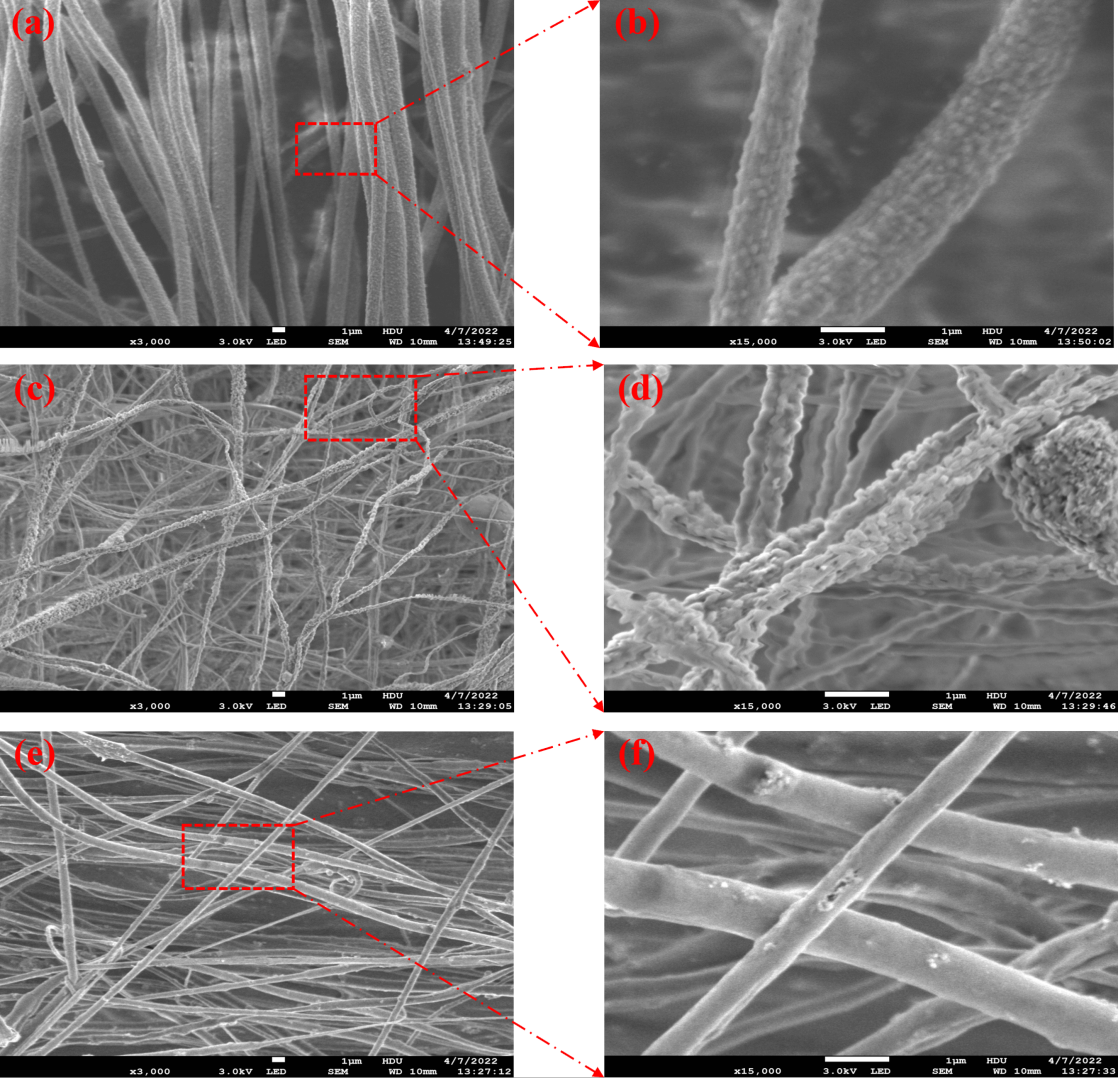
(a) SEM image of the P(VDF-TrFE) film, (b) enlarged part of (a), (c) SEM image of the P(VDF-TrFE)/ZnO film, (d) enlarged part of (c), (e) SEM image of the P(VDF-TrFE)/ZnO/GR film, and (f) enlarged part of (e).

The thickness of the piezoelectric film was determined to be approximately 120 μm using a Vernier caliper with a precision of 0.02 mm. The SEM images in Figure 8 reveal that the microstructure of the nanofilms consists of fibrous filaments at the micro/nanoscale. SEM images of a pure PVDF film in Figure 8a and Figure 8b exhibit filamentous fibers with a relatively smooth surface. In contrast, Figure 8c and Figure 8d show that the addition of ZnO to the P(VDF-TrFE) filaments leads to a rough and granular surface, caused by the aggregation of ZnO particles that embed onto the filament surface. By examining Figure 8e and Figure 8f, we observe that trace amounts of GR effectively inhibit the aggregation of ZnO particles, resulting in a smoother surface of P(VDF-TrFE)/ZnO/GR nanofilm filaments compared to P(VDF-TrFE)/ZnO.

The average fiber diameters of P(VDF-TrFE), P(VDF-TrFE)/ZnO, P(VDF-TrFE)/ZnO/GR were calculated as 1.23, 0.78 and 0.57 μm, respectively (Table 1). The addition of an appropriate amount of ZnO filler can improve the conductivity of the electrospinning solution, which leads to an increased stretching of the fiber filaments under the high voltage and to a reduced filament diameter. Moreover, since GR has a sheet-like structure with good electrical conductivity, adding a trace amount of GR material can further enhance the solution's conductivity and promote the dispersion of ZnO particles, resulting in finer and smoother nanofiber filaments.

**Table 1:** Diameter of fibers for different filler films.

Material	P(VDF-TrFE)	P(VDF-TrFE)/ZnO	P(VDF-TrFE)/ZnO/GR

fiber diameter (μm)	1.23	0.78	0.57

### Acoustic-electric conversion performance

**Figure 9:**
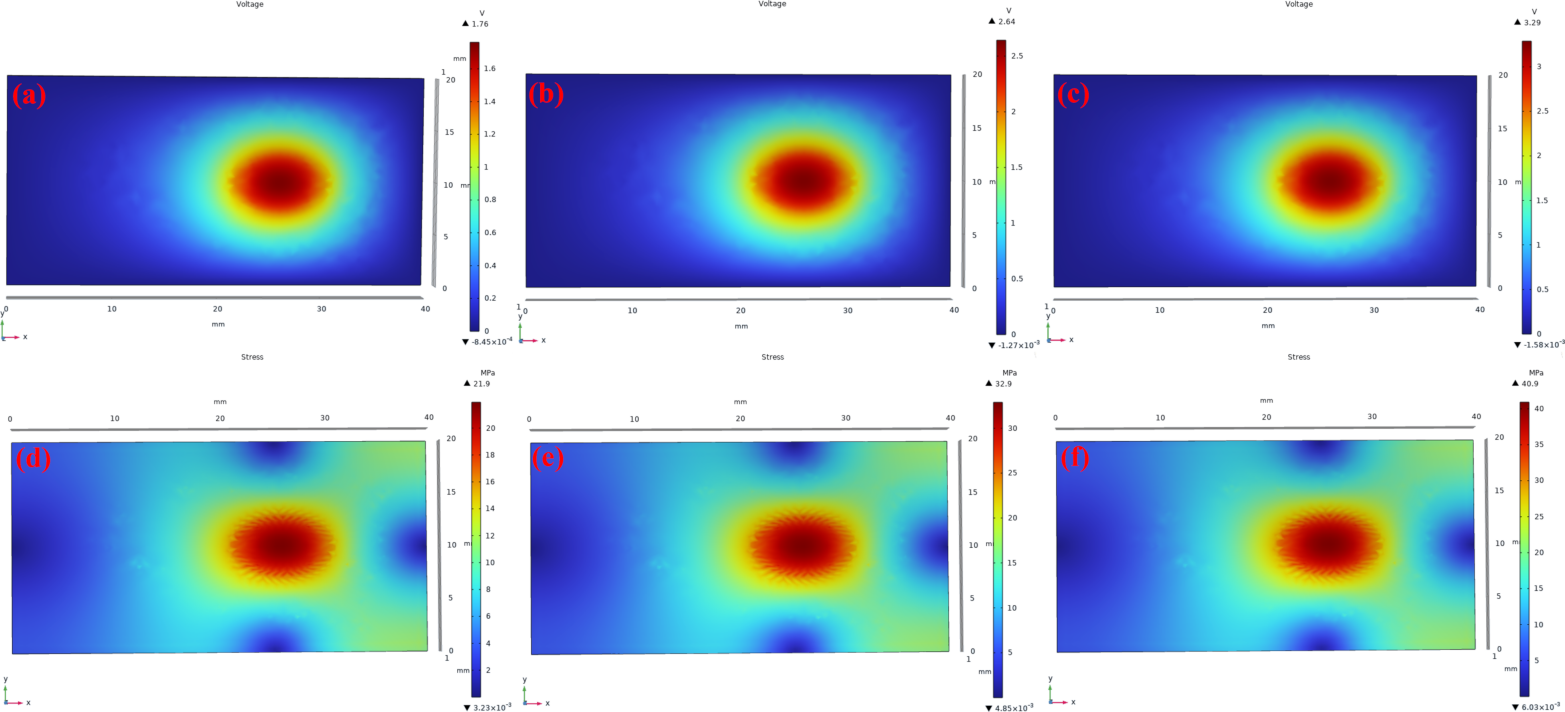
(a–c) Voltage distribution and (d–f) stress distribution in the piezoelectric film at sound pressures of (a, d) 65, (b, e) 75, and (c, f) 85 dB.

In order to further validate theoretically the effectiveness of the experiment, we conducted simulations using COMSOL software to analyze the internal voltage distribution and stress distribution of the piezoelectric film. Figure 9a–f illustrates the results of the simulations. Specifically, Figure 9a–c shows the simulated voltage distribution within the P(VDF-TrFE)/ZnO/GR piezoelectric film under sound pressures of 65, 75, and 85 dB. Figure 9d–f presents the corresponding stress distribution in the piezoelectric film. It is evident that as the sound pressure increases from 65 to 85 dB, the force exerted on the surface of the piezoelectric film also increases. This leads to a corresponding increase in the peak voltage within the film, which rises from 1.76 to 3.29 V. Additionally, the stress distribution within the film exhibits an upward trend, rising from 21.9 to 40.9 MPa. These findings further support the notion of a correlation between sound intensity, voltage generation, and stress distribution in the piezoelectric film.

**Figure 10:**
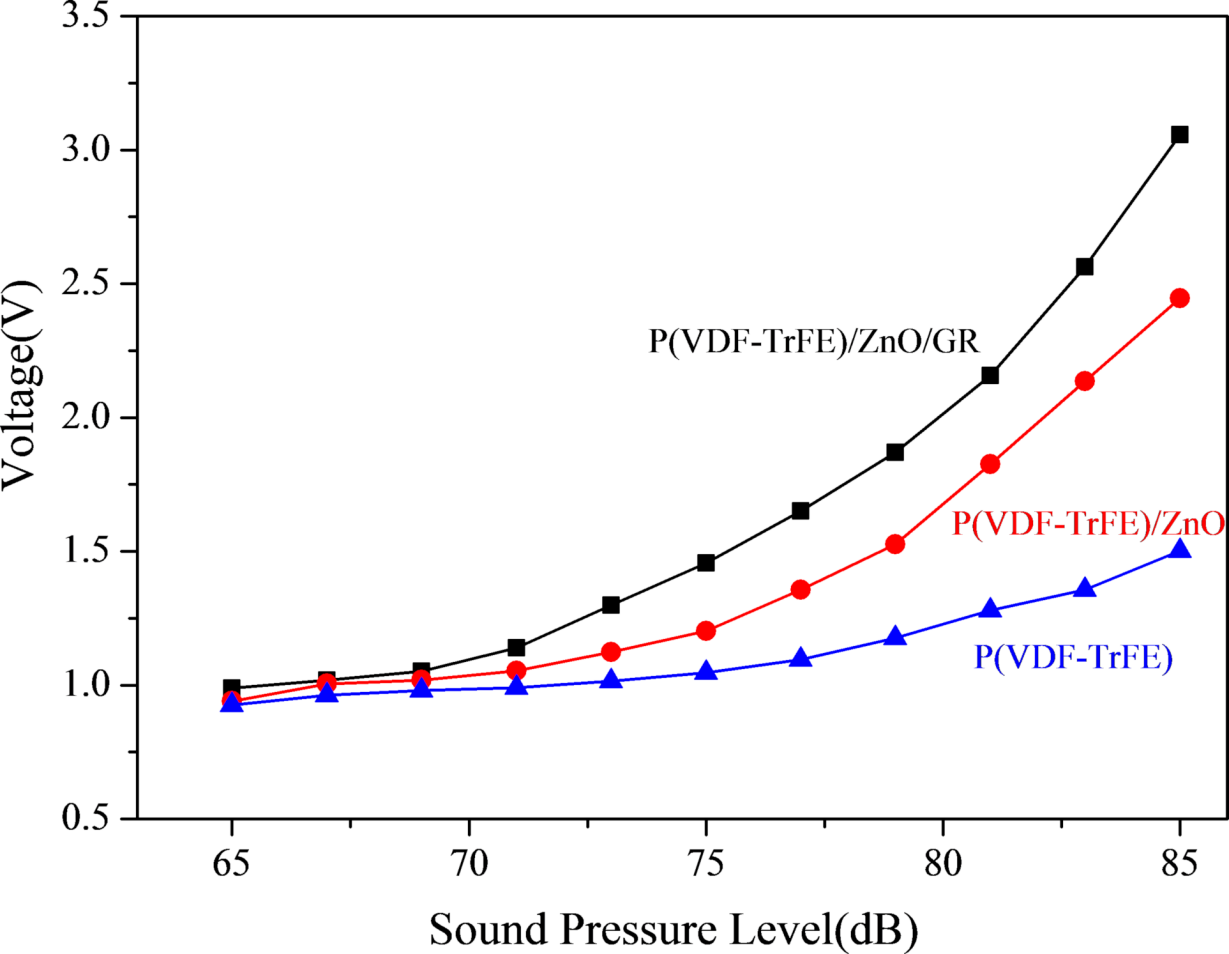
Voltage response diagram of the sensor at a sound frequency of 180 Hz and different sound pressures from 65 to 85 dB.

The heart sound signal consists of the first heart sound and the second heart sound, with a frequency range of 20 to 200 Hz [32–33]. This signal is characterized by low sound pressure and medium-to-high frequency, and the heart sound sensor must exhibit good acoustic and electrical response frequency bandwidth in the range of 20 to 200 Hz. To evaluate the sensor's acoustic-electric conversion ability at the same frequency but at varying sound pressure levels, we set the signal generator to emit a sine wave with a frequency of 180 Hz, and adjust the amplitude of the signal generator and power amplifier to control the sound pressure. We gradually increased the sound pressure from 65 to 85 dB, and measured the sensor response at 2 dB intervals, with the charge amplifier amplification set to 100 pC/V. To demonstrate that the P(VDF-TrFE)/ZnO/GR piezoelectric film has stronger acoustic-electric conversion performance than the P(VDF-TrFE)/ZnO piezoelectric film and the pure P(VDF-TrFE) piezoelectric film, we used these three films and the P(VDF-TrFE)/ZnO piezoelectric film with 10% ZnO and 0.1% GR as test samples. Figure 10 shows the results, with the response open-circuit voltage of the sensor gradually increasing with rising sound pressure, and the slope of the tangent line also increasing. The P(VDF-TrFE)/ZnO/GR sensor had higher response open-circuit voltage peaks than the P(VDF-TrFE) and P(VDF-TrFE)/ZnO sensors, indicating that the composite piezoelectric nanofilm with added ZnO and trace GR fillers showed stronger improvement in acoustic-electric conversion performance. This makes it more suitable for sound energy collection.

**Figure 11:**
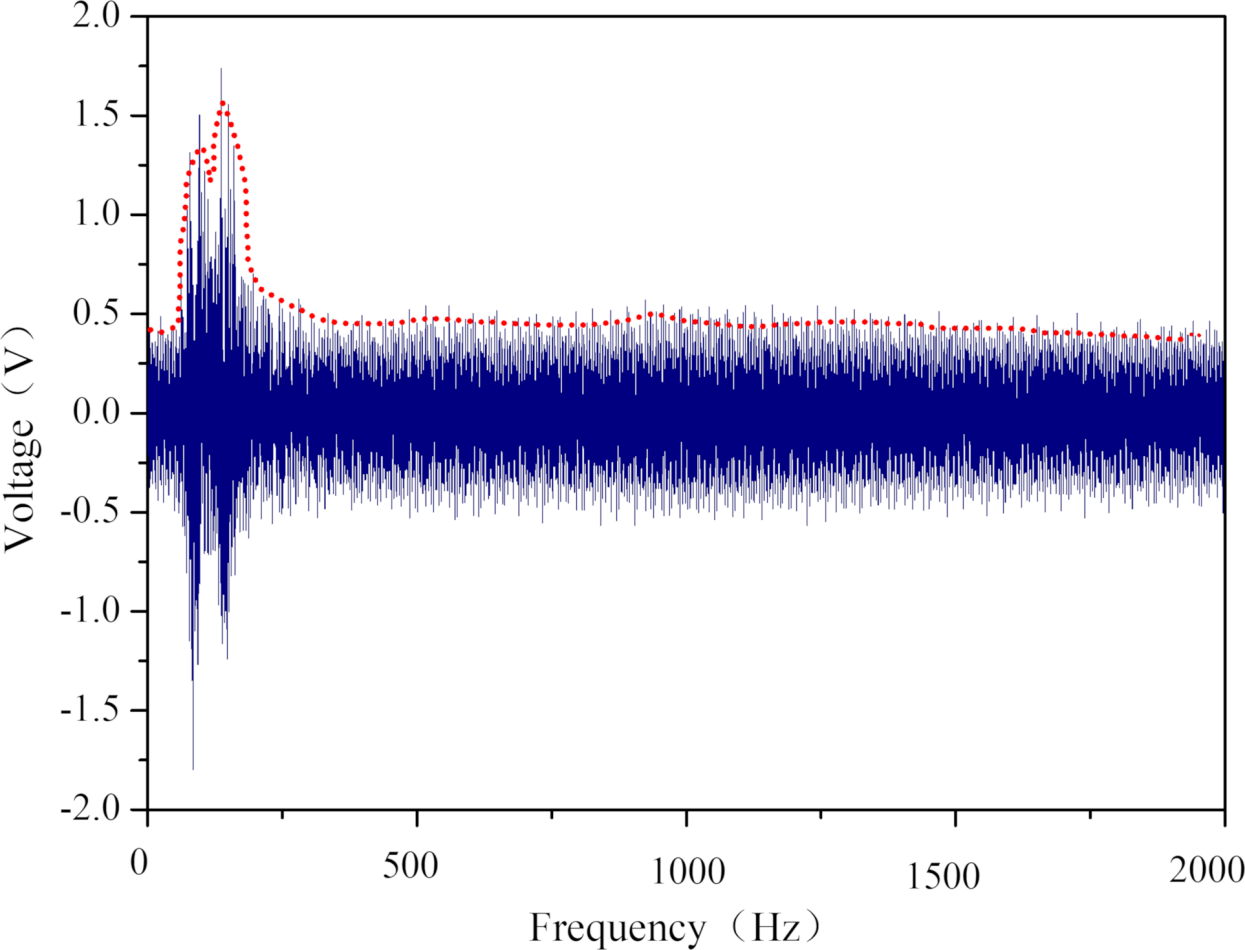
Sensor response at frequencies from 0 to 2 kHz at 65 dB sound pressure.

Heart sound signals are characterized by low sound pressure and medium-to-high frequency components. A heart sound sensor should have good acoustic-electric conversion capability in the frequency range of heart sound signals. A signal generator was used to produce a sine wave with a frequency range of 0 to 2000 Hz, with a frequency sweep step of 100 Hz/s. The sound pressure was set to 65 dB, and the charge amplifier's gain was set to 100 pC/V. Figure 11 shows the voltage peaks recorded by the oscilloscope at different times during the frequency sweep. The peak open-circuit voltage significantly increases between 50 and 200 Hz at a fixed sound pressure of 65 dB.

**Figure 12:**
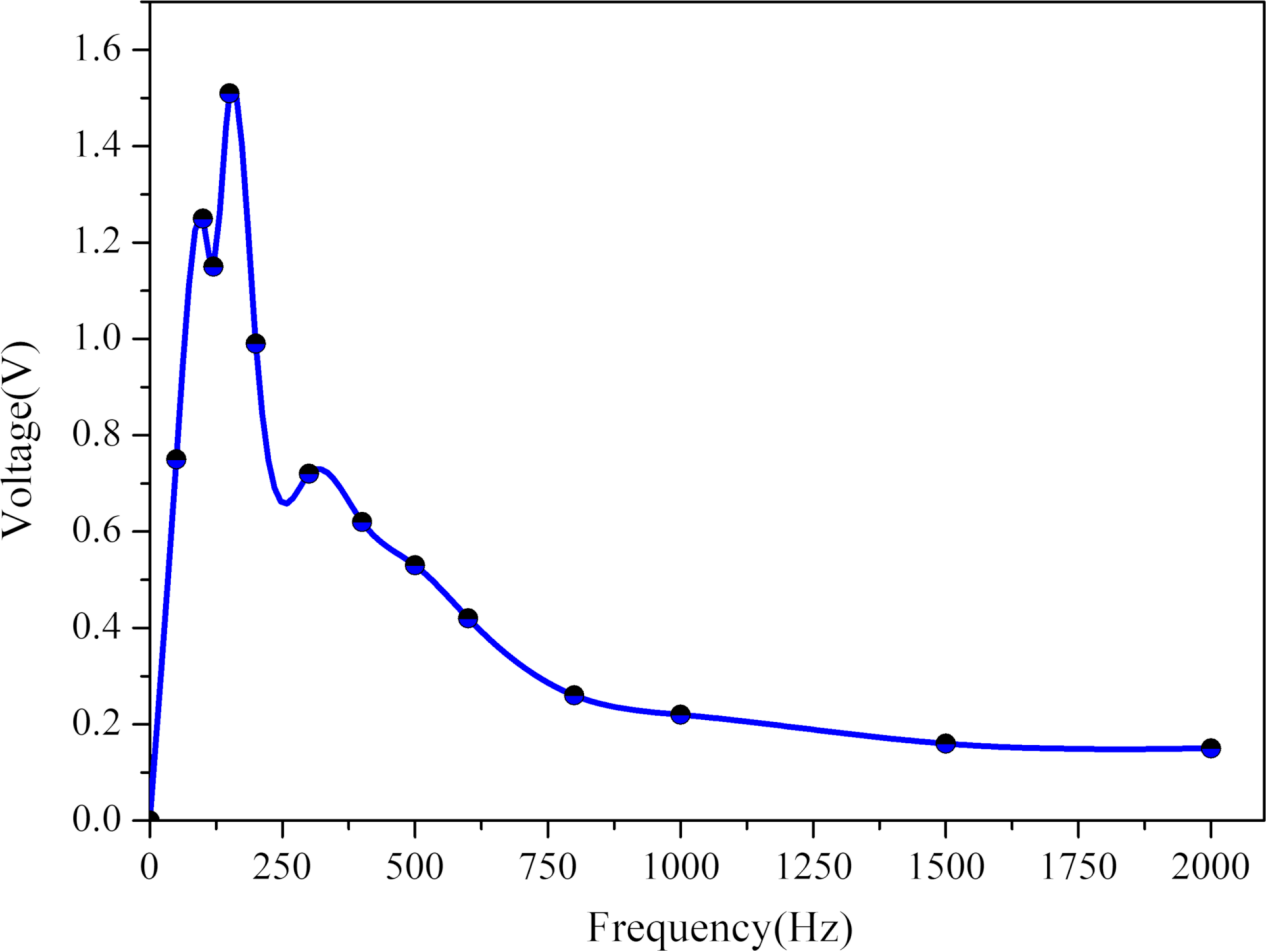
Different frequency voltage response diagram of the sensor at 65 dB sound pressure.

**Figure 13:**
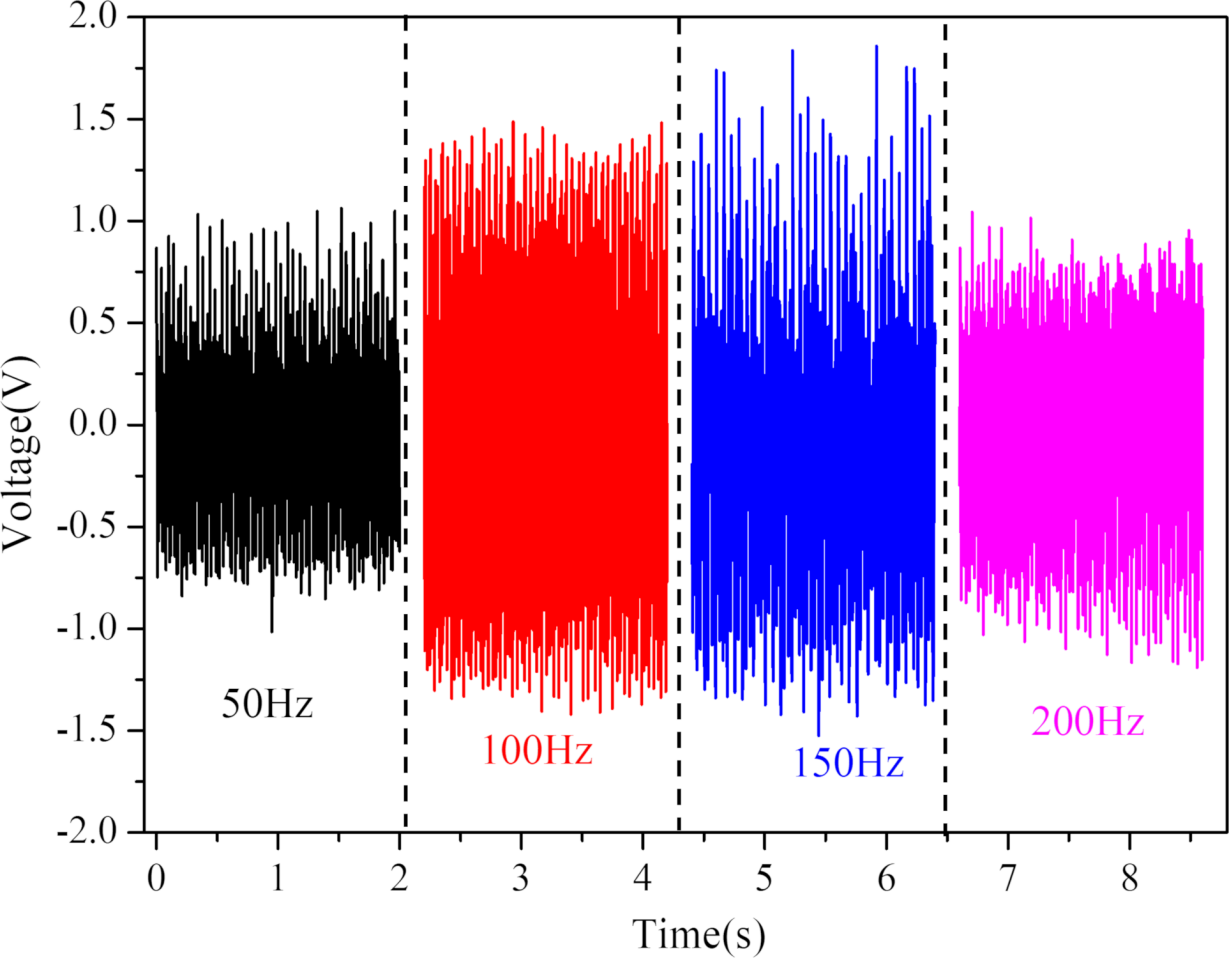
Open-circuit voltage plot of the sensor at 65 dB sound pressure at different frequencies.

To further verify the acoustic-electric conversion performance of the sensor, the experiment measured its peak open-circuit voltage at 14 different frequencies, as depicted in Figure 12, with a fixed sound pressure of 65 dB. The sensor developed in this study has a wide frequency response range, with excellent acoustic-electric response performance in the frequency range of 20 Hz (the first test point) to 200 Hz (the sixth test point), satisfying the frequency response requirements of a heart sound sensor. Figure 13 illustrates the open-circuit voltage response of the sensor at different frequencies under a 65 dB sound pressure, showing a symmetrical voltage response about the 0 V line, with clear acoustic-electric response characteristics.

The piezoelectric coefficient (*d*_33_) is a crucial parameter for assessing the performance of piezoelectric devices. A higher value of the piezoelectric coefficient signifies superior device performance [34]. In the context of energy harvesting from piezoelectric films, Equation 2 defines the relationship between the relative charge (*Q*) generated on the surface of the piezoelectric material, with an area (*S*), and the applied mechanical stress (Δ*F*) [35]:


[2] $$Q=d_{33}\times S\times \Delta F.$$

Under short-circuit conditions, the current can be expressed by Equation 3 [35]:


[3] $$I=\frac{\Delta Q}{\Delta t}=d_{33}\times S\times \frac{\Delta F}{\Delta t}.$$

**Figure 14:**
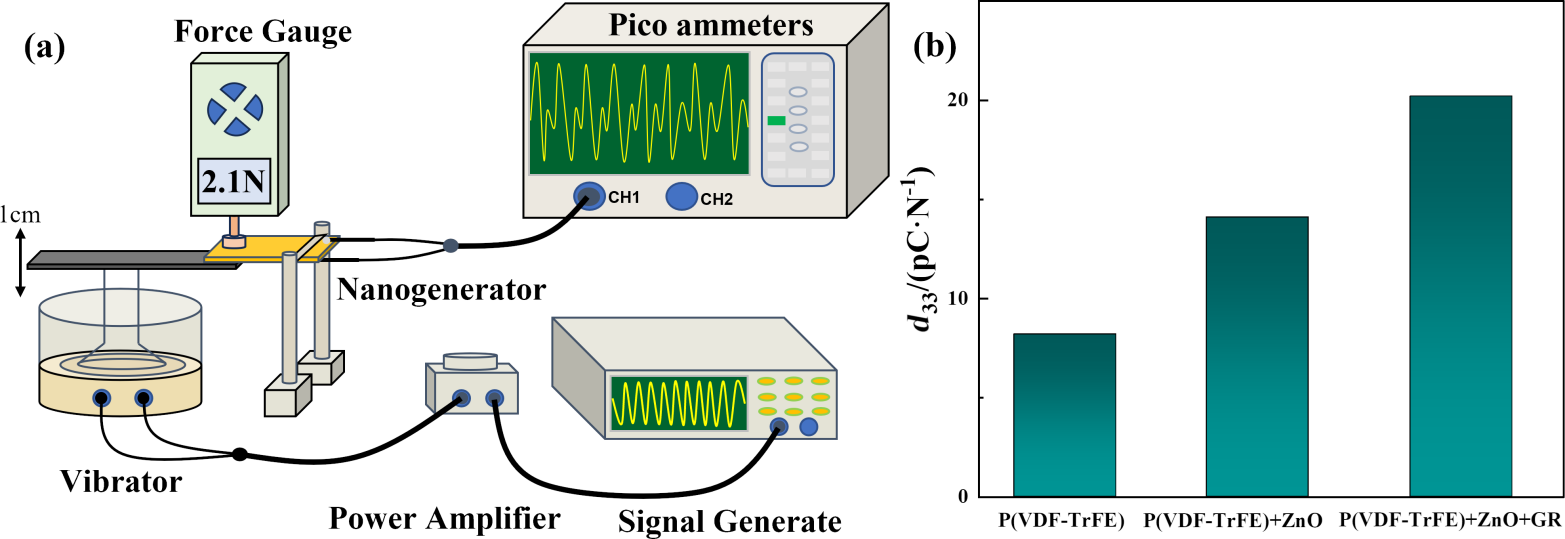
(a) Piezoelectric coefficient measurement system and (b) results of the piezoelectric coefficient.

To determine the piezoelectric coefficient of the piezoelectric film, a dynamic measurement method was utilized in this study. The piezoelectric film was exposed to periodic forces (Δ*F*) applied by the excitation system under short-circuit conditions, leading to the generation of an electric current. The piezoelectric coefficient can be calculated using Equation 3. The self-constructed piezoelectric coefficient measurement system is illustrated in Figure 14a, while the corresponding results of the piezoelectric coefficient are presented in Figure 14b.

### KNN heart sound classification recognition algorithm

**Figure 15:**
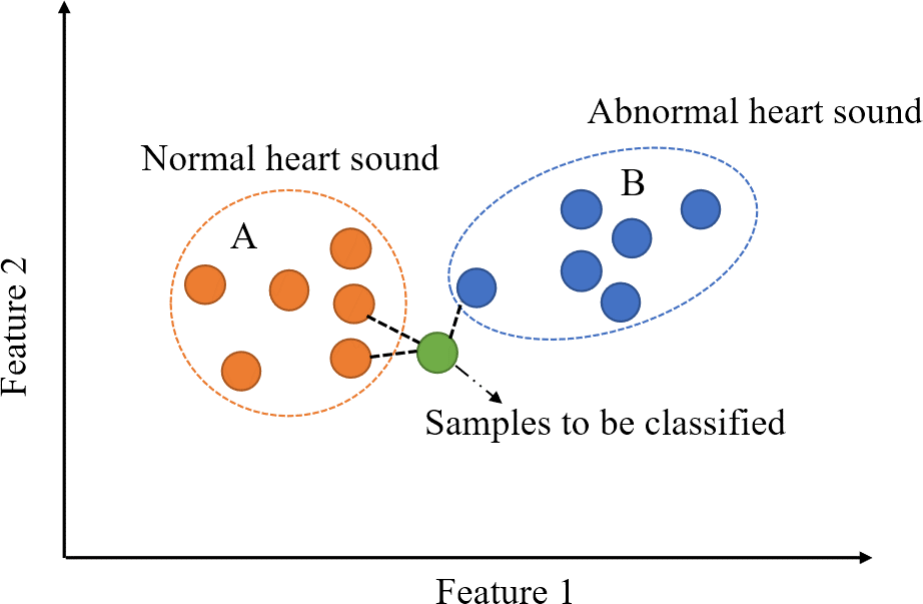
Example of KNN classification algorithm.

The KNN classification algorithm is widely used in machine learning, and its fundamental classification idea is that a sample in a feature space belongs to the same category as the majority of its *K* nearest neighbors and that this sample has the same characteristics as this class [36]. Since KNN mainly relies on a finite number of neighboring samples to determine the category characteristics, this method is more suitable for sample sets with more overlap or overlap of the class domain than other methods. The factors affecting the classification effect of KNN mainly lie in the selection of the *K* value and the distance formula. In Figure 15, an example is provided for two-dimensional heart sound data feature space. *K* takes a value of 3, and two of the three nearest neighbors of the sample to be classified belong to class A, while one belongs to class B. Hence, the sample belongs to category A, which means it is a normal heart sound. However, when *K* = 1, the sample being classified belongs to class B, meaning it is judged to be an abnormal heart sound. From this point of view, the selection of the *K* value is the focus of constructing a KNN heart sound classification model. In this paper, the Euclidean distance calculation formula shown in Equation 4 is selected, and the most appropriate *K* value is determined by cross-validation:


[4] $$d\left(x,y\right)=\sqrt{\sum_{i=1}^{n}{\left(x_{i}-y_{i}\right)}^{2}},$$

where *d* represents the distance. *x*_i_ and *y*_i_ represent the coordinates.

**Figure 16:**
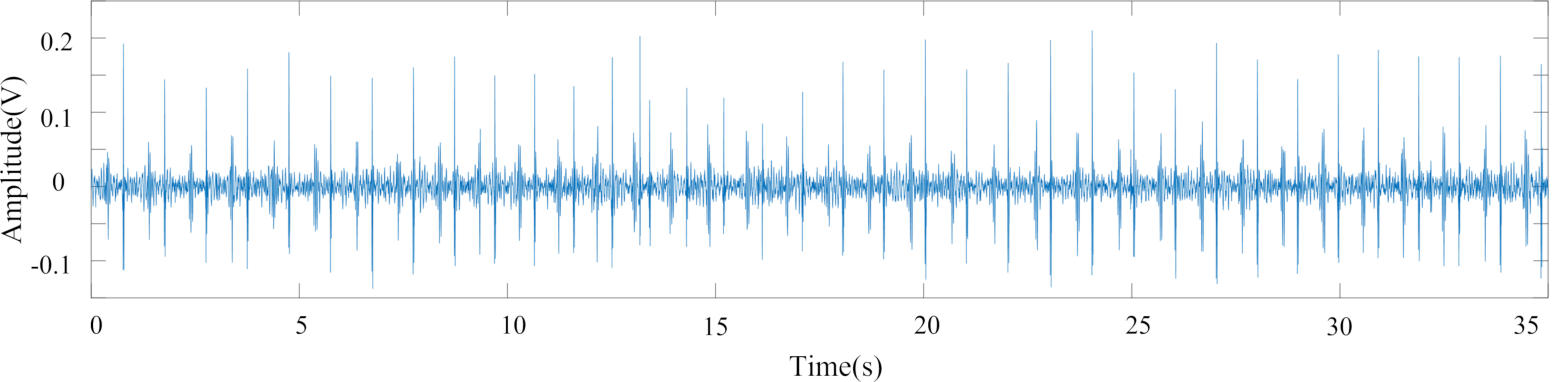
Heart sound time domain diagram before pretreatment. Figure 16 contains information from [PhysioNet/CinC Challenge 2016 database] which is made available under the Open Data Commons Attribution License v1.0, https://opendatacommons.org/licenses/by/1-0/.

**Figure 17:**
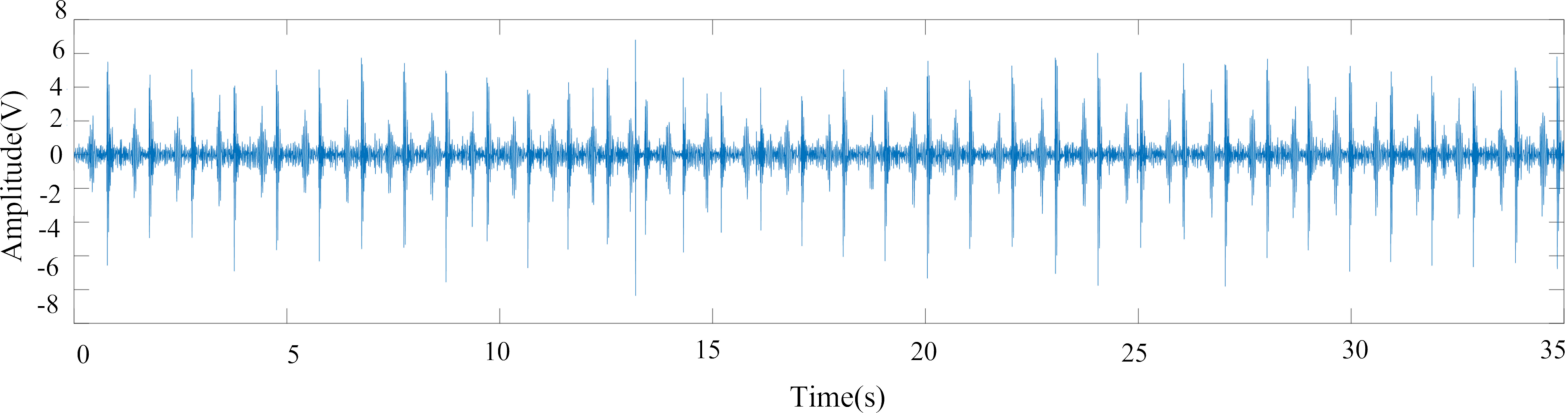
Heart sound time domain diagram after pretreatment. Figure 17 contains information from [PhysioNet/CinC Challenge 2016 database] which is made available under the Open Data Commons Attribution License v1.0, https://opendatacommons.org/licenses/by/1-0/.

The heart sound dataset chosen for the experiment consists of 65 sets of normal heart sound data collected using homemade heart sound sensors and Physionet open-source data [37]. The 65 sets of heart sound data were collected from 13 adult human samples with normal heart sounds, and saved in WAV format using the experimental self-built heart sound acquisition system. The heart sound data in the PhysioNet/Computing in Cardiology Challenge 2016 (PhysioNet/CinC Challenge 2016) database includes 2461 normal heart sounds and 665 abnormal heart sounds in WAV format. All normal heart sounds are labeled “−1”, and all abnormal heart sounds are labeled “1”. As the heart sound acquisition environment in the database is different, the collected heart sound signal may contain electromagnetic interference, lung sounds, and other noise. In the experiment, a 20 to 200 Hz band-pass filter and 50 Hz notch filter were generated using MATLAB's built-in “fdatool” tool, both of which use a third-order Butterworth filter. Since the amplitude of heart sound signals varies greatly due to the different devices used for collecting heart sound database data, the Z-Score function in MATLAB was employed to standardize the data signals separately. This yielded signal indicators of the same magnitude, increasing the comparability between data. Figure 16 shows a time domain diagram of a raw heart sound signal (normal heart sound) in the heart sound database, while Figure 17 illustrates the time domain diagram of the preprocessed heart sound signal. Comparing the waveforms of the two figures, it can be observed that the characteristics of the first heart sound and the second heart sound of the preprocessed heart sound signal are more apparent, the noise removal is better, and the amplitude of the heart sound signal is improved.

**Figure 18:**
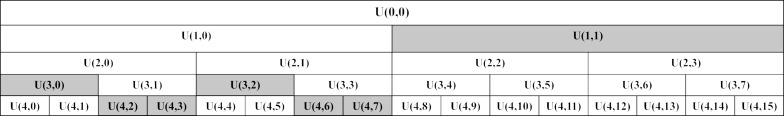
Wavelet packet decomposition and optimal basis selection diagram.

In this experiment, 34 heart sound features were selected, including 14 time domain features, 13 mel frequency cepstral coefficient (MFCC) features, and seven wavelet features. Table 2 displays the selected time and frequency domain features. MFCC is a speech feature that mimics the sensitivity of sound signals of different frequencies in the human ear, based on the hearing mechanism. Extracting MFCC features is useful for modeling heart sound signals. The wavelet feature extraction method uses “db6 wavelet decomposition” to generate seven feature vectors. The “db6 wavelet decomposition” decomposes the heart sound signal into five layers, selects seven optimal bases of the heart sound signal according to the filtering characteristics of the binary wavelet sub-band, and reconstructs feature vectors. As shown in Figure 18, the shaded background annotation represents the seven optimal bases that have been selected.

**Table 2:** The 14 selected heart sound signatures in the time and frequency domains.

Number	Feature

1	mean value characteristics of heart sound signal data
2	median characteristics of heart sound signal data
3	standard deviation characteristics of heart sound signal data
4	characteristics of mean absolute deviation of heart sound signal data
5	quantile characteristics of heart sound signal data (25%)
6	quantile characteristics of heart sound signal data (75%)
7	4th percentile difference of heart sound signal data
8	deviation of heart sound signal data
9	kurtosis of heart sound signal data
10	Shannon entropy of heart sound signal data
11	spectral entropy of heart sound signal data
12	frequency characteristics of heart sound signal data: dominant frequency, dominant frequency ratio, and dominant frequency amplitude
13	
14	

In this experiment, the “Classification Learner toolbox” in MATLAB was used to train a heart sound classification model. This toolbox allowed us to explore supervised machine learning by selecting various classifiers, exploring data, selecting features, specifying validation scenarios, training models, and evaluating the results. Automated training was used to search for the best classification model types, including decision trees, support vector machines, logistic regression, and KNN. After cross-validation, a fine KNN (with a *K* value of 1) was chosen as the heart sound classification model.

The verification method used is cross-validation. The heart sound data is divided into five equal copies, where four copies are utilized for model training and one copy is used for validation. The training and validation are alternately rotated for five times, and the average of the five validation results is computed to measure the accuracy of the model. The recognition rate is adopted as the metric to assess the quality of the classification model, and was calculated using Equation 5:


[5] $$\text{accuracy}=\frac{\text{TP}+\text{TN}}{\text{TP}+\text{TN}+\text{FP}+\text{FN}}.$$

The term “TP” represents the number of heart sounds that were correctly identified as normal, while “FN” represents the number of normal heart sounds that were mistakenly classified as abnormal. “TN” represents the number of heart sounds with abnormal sounds that were correctly identified as such, and “FP” represents the number of abnormal heart sounds that were mistakenly classified as normal. After verification, the heart sound classification model trained in the experiment achieved an accuracy rate of 94.8%.

**Figure 19:**
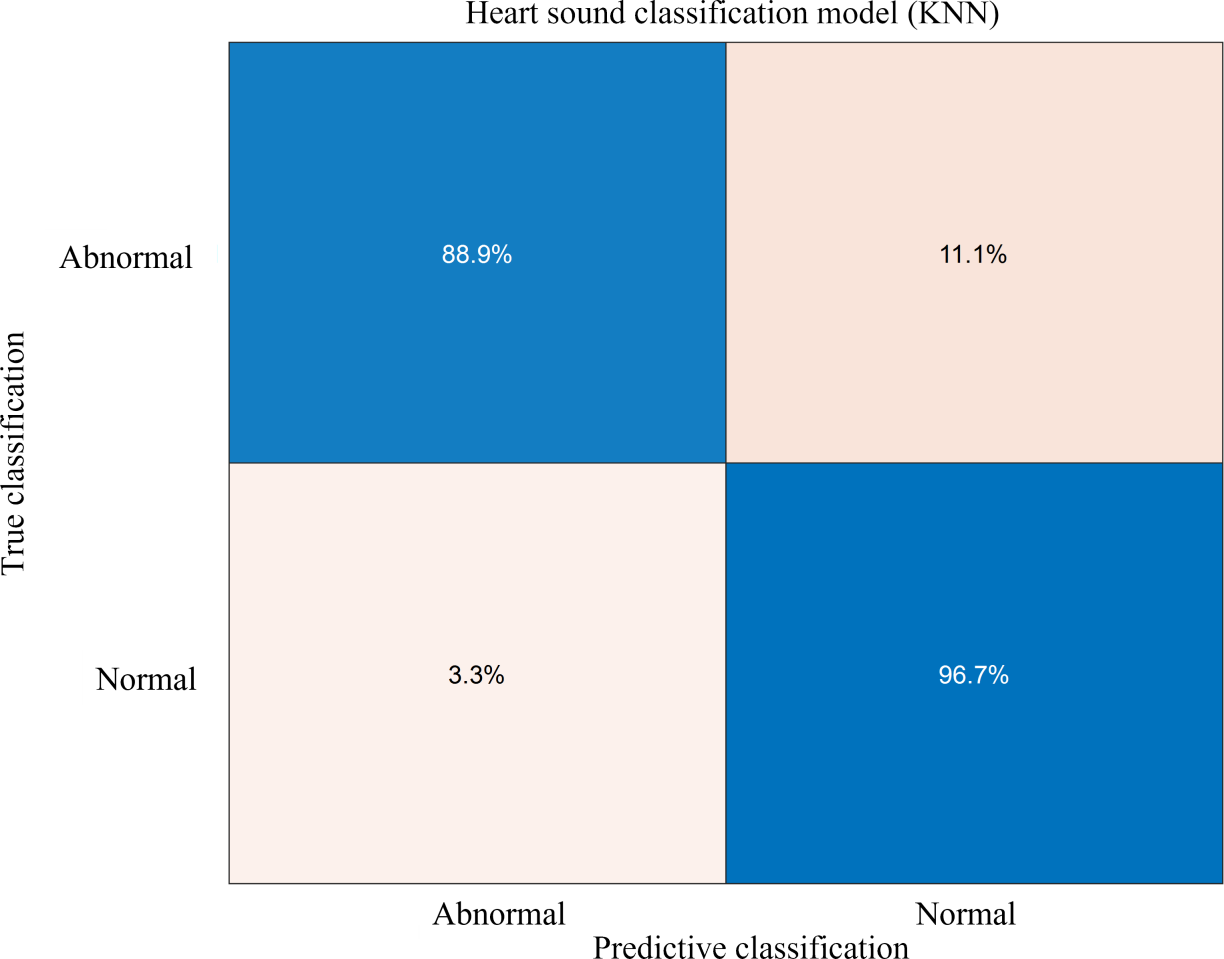
Validation confusion matrix of heart sound classification model (fine KNN).

According to Liu et al. [38], adaptive noise-complete empirical modal decomposition permutation entropy combined with a support vector machine was used to classify normal and abnormal heart sound samples in the PhysioNet/CinC Challenge 2016 database, and the classification accuracy was 87%. Hence, the KNN heart sound classification model has a higher accuracy for the classification of normal and abnormal heart sound signals. The confusion matrix of the heart sound classification model is shown in Figure 19. The matrix indicates that this classification model has an 11.1% probability of misclassifying abnormal heart sound signals as normal heart sounds and a 3.3% probability of misclassifying normal heart sound signals as abnormal heart sounds. This might be due to the fact that there are more normal heart sounds than abnormal heart sounds in the heart sound signal data. Therefore, further supplementation of heart sound data is needed to optimize the heart sound classification model. Yet, the model trained in this paper can accurately predict normal and abnormal heart sounds. When combined with the heart sound acquisition system built in the previous section, it can fulfill the entire process from heart sound collection to heart sound diagnosis, thereby providing a new solution for the study of heart disease.

## Conclusion

In this paper, P(VDF-TrFE)/ZnO/GR flexible piezoelectric composite films with excellent acoustic-electric conversion performance were prepared using the electrospinning process. Electron microscopy revealed that the filaments of P(VDF-TrFE)/ZnO/GR films had finer and smoother characteristics than those of P(VDF-TrFE)/ZnO flexible piezoelectric composite films without GR. Analysis of X-ray diffraction patterns indicated that the β-phase content of P(VDF-TrFE)/ZnO/GR piezoelectric composite films was higher than that of P(VDF-TrFE)/ZnO and P(VDF-TrFE). P(VDF-TrFE)/ZnO/GR was packaged into a flexible piezoelectric nanoscale heart sensor, and the outermost layer of silica gel effectively protected the flexible nanofilm and adhered to the skin. In this paper, the acoustic-electric conversion performance of P(VDF-TrFE)/ZnO/GR piezoelectric composite films was evaluated using a self-built acoustic-electric conversion performance test platform. Experimental comparisons revealed that the P(VDF-TrFE)/ZnO/GR piezoelectric composite films exhibit superior acoustic-electric conversion performance in comparison to P(VDF-TrFE) piezoelectric films. Frequency sweeping experiments revealed that the experimentally prepared P(VDF-TrFE)/ZnO/GR piezoelectric composite nanofilms exhibit excellent acoustic-electric conversion performance of low-frequency and medium-frequency sound signals, meeting the requirements for good acoustic-electric conversion ability in the frequency range of heart sound signals.

In this paper, a KNN heart sound classification model was trained using MATLAB. Additionally, a heart sound acquisition platform was constructed using LabVIEW, which calls the MATLAB script to input real-time heart sound signals into the trained heart sound classification model for predicting normal and abnormal heart sounds.

The wearable flexible nanoscale heart sound sensor, combined with the self-built heart sound acquisition system, is capable of predicting the normality of real-time heart sound signals. In comparison to traditional heart sound stethoscopes, this sensor offers the advantages of being soft and close to the skin, while the use of a machine learning algorithm to classify heart sound signals solves the shortcomings of traditional auscultation, such as introducing murmurs and relying on personal experience for interpretation.
